# Addressing the COVID-19 Mental Health Crisis: A Perspective on Using Interdisciplinary Universal Interventions

**DOI:** 10.3389/fpsyg.2021.644337

**Published:** 2021-04-13

**Authors:** Geraldine Przybylko, Darren Peter Morton, Melanie Elise Renfrew

**Affiliations:** Lifestyle Medicine and Health Research Centre, Avondale University College, Cooranbong, NSW, Australia

**Keywords:** universal, mental health, positive psychology, lifestyle medicine, digital, multicomponent, interdisciplinary

## Abstract

Mental health is reaching a crisis point due to the ramifications of COVID-19. In an attempt to curb the spread of the virus and circumvent health systems from being overwhelmed, governments have imposed regulations such as lockdown restrictions and home confinement. These restrictions, while effective for infection control, have contributed to poorer lifestyle behaviors. Currently, Positive Psychology and Lifestyle Medicine are two distinct but complimentary disciplines that offer an array of evidence-based approaches for promoting mental health and well-being across a universal population. However, these strategies for improving mental health are typically used in isolation. This perspective calls for a new paradigm shift to create and rollout well-designed interdisciplinary universal multicomponent mental health interventions that integrates the benefits of both disciplines, and uses innovative digital mental health solutions to achieve scalability and accessibility within the limitations and beyond the COVID-19 lockdown and restrictions.

## Introduction

The prevalence of depression and anxiety is estimated at 586 million people worldwide (World Health Organization, 2017), with an annual economic burden of US$1 trillion, and less than one mental health professional for every 10,000 people (United Nations, 2020). This bleak backdrop predated the Coronavirus Disease 2019 (COVID-19) outbreak, of which 111 million cases and 2.5 million deaths have been reported as at 21 February 2021 (CNN health, 2021).

In an attempt to curb the spread of COVID-19 and circumvent health systems from being overwhelmed, governments have imposed regulations such as wearing masks, lockdown restrictions, social distancing, travel bans, and home confinement resulting in isolation (Dubey et al., 2020). These restrictions, while effective for infection control, have contributed to poorer lifestyle behaviors, such as physical and social inactivity, poor sleep quality, and unhealthy eating patterns (Ammar et al., 2020a). Unremarkably, poorer mental health has been observed within the general population, with health workers and COVID-19 sufferers being especially impacted (Deng et al., 2020; Pera, 2020; Rossi et al., 2020; Shi et al., 2020; Bueno-Notivol et al., 2021; CNN health, 2021). For example, a meta-analysis of 43 studies indicated that anxiety in the general population was at least 3 times higher during COVID-19 than before the pandemic (Santabarbara et al., 2020). Similarly, in the United States, Ettman et al. (2020) found 1 in 2 people reported depressive symptoms during COVID-19, which was three times higher than pre-pandemic circumstances. In Australia, Fisher et al. (2020) found that mental health issues had at least doubled in the first month of COVID-19 restrictions.

Children and adolescents have also been affected during COVID-19 due to factors such as school closures and diminished opportunities for social interaction and connection (Cuartas, 2020). In a rapid review of 63 studies, Loades et al. (2020) observed higher rates of depression and anxiety during and after isolation. The authors encouraged preventative support and early intervention for children and adolescents to combat the increasing mental health issues. At the other end of the age spectrum, a number of studies have indicated that older adults are disproportionally affected by COVID-related mental health disorders, morbidity and mortality (Palmer et al., 2020; Zheng et al., 2020; Bae et al., 2021; Tsamakis et al., 2021).

Preserving the mental health and resilience of our population and already burdened health workers is paramount to ensuring global recovery from the pandemic (Santarone et al., 2020; Sharma and Verma, 2020). Hence, addressing the unprecedented mental health crisis must be an integral part of a comprehensive public health response to the COVID-19 pandemic and should involve the deployment and resourcing of: (1) population-based (universal) interventions to reduce the development of mental health disorders; (2) selective interventions to address those who are at high risk; and (3) clinical interventions to treat those who are suffering (Fisher et al., 2020). Currently, universal interventions are not commonly utilized, despite their potential to promote the mental health and resilience of all people. Keyes and Galea (2016) insightfully contend that when one reviews the health initiatives of the past century that have most significantly improved the lives of the population, only a small number are due to precise and personalized medicine. Hence, there are a number of calls for universal mental health interventions to mitigate the emerging mental health crisis due to COVID-19. This perspective article argues that widespread benefits can be achieved through: (1) the implementation of interdisciplinary universal mental health interventions; and (2) multicomponent interventions will likely confer greater benefits than a single modality approach.

## Universal Mental Health Interventions

Universal, or population-based, mental health interventions, are defined as non-clinical, primary prevention strategies directed at an entire population that address generic mental health risk and protective factors (Purtle et al., 2020; Rudd and Beidas, 2020). Universal mental health interventions aim to encourage general mental wellness through the promotion of psychoeducation and messages that destigmatize mental disorders, and deliver essential support to maintain psychological well-being. The literature suggests there are a number of benefits associated with universal mental health interventions including: the potential to reduce numerous risk factors; typically a lower cost per person; the ability to reach individuals who do not reach a diagnostic threshold; and a reduction in the stigma associated with mental health issues (Greenberg and Abenavoli, 2016).

While potential disadvantages of universal mental health interventions have been identified, including time and resources spent on participants who may not develop the adverse outcomes (Greenberg and Abenavoli, 2016) and the effect sizes tending to be smaller than selective interventions (Tan et al., 2014; Deady et al., 2017), there is a strong case for their benefits. For example, while effect sizes may be smaller, the impact can be large when translated to an entire population (Tan et al., 2014). Examples of successful, high-impact universal interventions include: vaccinations to reduce the spread of viruses; tobacco policy changes to reduce smoking-related mortality; mandatory use of seatbelts to reduced motor vehicle mortality (Sampson and Galea, 2018); and social distancing, the use of masks, hand washing and quarantine, to reduce the spread of the COVID-19, as previously mentioned. According to the Centers of Disease Control and Prevention, universal mental health interventions are relatively new, however, this approach is needed more than ever during COVID-19 (Evans and Bufka, 2020).

While there is a good case for universal mental health interventions, we argue that better outcomes can be achieved though interdisciplinary universal mental health interventions. Interdisciplinary interventions are characterized by the incorporation of evidence-based strategies and initiatives drawn from two or more disciplines. Specifically, we suggest that efficacious universal mental health interventions can be created by merging the disciplines of Positive Psychology and Lifestyle Medicine. These disparate but complimentary disciplines are providing an array of evidence-based approaches for promoting mental health and well-being across a universal population.

## Positive Psychology and Mental Health

Prior to the late 1990s, psychology was almost exclusively predicated on a deficit model, focusing on mental illness such as depression, stress, anxiety, and dysfunctional attitudes and behaviors (Kobau et al., 2011). During his time as president of the American Psychological Association in the late 1990s, Martin Seligman formalized the field of “Positive Psychology,” characterized by the study of: positive emotions; positive character traits; optimal functioning; and empowering a state of “flourishing” for individuals, groups and institutions (Seligman et al., 2005). Over the past two decades Positive Psychology has experienced exponential growth in its size, reach, and impact (Rusk and Waters, 2013), and its influence continues to expand beyond the psychology discipline to education, psychiatry, neuroscience, health, and business (Rusk and Waters, 2013; Seligman, 2019). Further, there has been a proliferation in its supporting evidence-base and the use of Positive Psychology Interventions (PPIs).

PPIs involve a variety of strategies designed to increase positive affect and life satisfaction, including: expressing gratitude (Cunha et al., 2019), focusing on what went well (Mongrain and Anselmo-Matthews, 2012), performing acts of kindness (Curry et al., 2018), practicing forgiveness (Wade et al., 2014), expressing humor (Wellenzohn et al., 2016) and identifying strengths (Ghielen et al., 2017). Carr et al. (2020) conducted a systematic review and meta-analysis of 347 studies of PPIs and reported significant small to medium effect sizes for improvements on: positive aspects of well-being (i.e., positive affect and life satisfaction), depression, anxiety and stress.

There are a limited number of studies that have examined the benefits of PPIs in the context of COVID-19 (Kavcic et al., 2020). A study of 1,059 adults in the United States found that sharing positive emotions prompted positivity resonance, resilience, and positive mental health during the pandemic (Prinzing et al., 2020). Despite a dearth of pandemic-based research, there are calls for Positive Psychology principles to be emphasized during COVID-19. For example, in a review of 34 meta-analyses, Fischer et al. (2020) argued that self-guided therapeutic approaches and Positive Psychology strategies (such as the expression of optimism, gratitude, and kindness) should be considered first-line interventions to be used during quarantine and social distancing to alleviate depression, anxiety and stress, and improve subjective well-being. Similarly, Waters et al. (2021) advocated for the incorporation of Positive Psychology knowledge, skills and practices (i.e., meaning, coping, self-compassion, courage, gratitude, character strengths, positive emotions, positive interpersonal processes, and high-quality connections) to support individuals by building positive processes and capacities to strengthen mental health during a pandemic. For practical Positive Psychology strategies refer to Table 1.

**Table 1:** Practical Positive Psychology and lifestyle recommendations during COVID-19.

**Strategy**	**Recommendation**	**Examples**	**Reference**
Positive Psychology	Expressing gratitude	* Use a gratitude journal * Daily affirm and recognize others in a face-to-face or virtual environment	Fischer et al. (2020)
	Performing acts of kindness	* Perform an act of kindness each day	Fischer et al. (2020)
	Self-compassion	* Treating yourself each day with the same compassion as you show your friends	Waters et al. (2021)
	Resilience	* Keep perspective about life and challenges * Maintain a good social support * Be hopeful about life	Waters et al. (2021)
Physical Activity	Increase physical activity	* Join exercise classes online * Walk up and down the stairs	World Health Organization (2020b)
	Increase muscle strength and balance training	* Resistance exercises	World Health Organization (2020b)
	Breaking prolonged sitting	* Stand up while working, talking on the phone or watching TV	World Health Organization (2020b)
	Home-based physical tests	* Stair climb test * Balance test * Sit-and-reach test	da Cunha de Sá-Caputo et al. (2020)
Healthy Diet	Consumer regular meals	* Greatest intake of energy taking place in the morning	Ammar et al. (2020b)
	Reducing meal frequency	* Avoid eating in between meals	Ammar et al. (2020b)
	Healthy food and drink intake	* Eating more whole plant-based foods * Stay hydrated * Avoid refined and high glycemic foods	Ammar et al. (2020b) Ammar et al. (2020b)
	Adapting intermittent or long fasting period	* Fast 12 hours or more from dinner to breakfast	Ammar et al. (2020b)
Sleep Hygiene	Regular waking up and night schedule	* Use natural circadian preference to guide sleep patterns * Bring some structure to the day especially for children	Altena et al. (2020)
	Avoid or minimize screen time in your bedroom	* Do not take screen devices and tablets into the bedroom * Alternatively, switch off devices and tablets to reduce sleep disruption such as light and noise pollution	Altena et al. (2020)
	Engage in physical exercise	* Exercise regularly, preference during the day	Altena et al. (2020)

It is enlightening that a positive approach to mental well-being can alleviate depression, anxiety and stress, and improve positive emotion as PPIs do not have the stigma that can be associated with clinical interventions. Hence, PPIs might be more appropriate when targeting a population-level (i.e., universal interventions), as non-clinical individuals might be more inclined to engage with them.

Importantly, it has been argued that multicomponent PPIs are more beneficial than single modality approaches (Morton et al., 2020; Przybylko et al., 2021). Hendriks et al. (2019) conducted a systematic review and meta-analysis of 50 RCTs and concluded that Multicomponent PPIs (MPPIs) were efficacious, with a small to moderate effect on psychological well-being and a small effect on subjective well-being. Hence, Sin and Lyubomirsky (2009) encouraged practitioners to use MPPIs in a “shotgun” approach, asserting it to be more engaging and effective than single modality interventions.

## Lifestyle Medicine and Mental Health

Like Positive Psychology, the discipline of Lifestyle Medicine is an evidence-based discipline that has emerged rapidly over the past two decades. Lifestyle Medicine seeks to “treat the cause” of chronic diseases and involves the application of lifestyle-based therapies such as positive nutrition, physical activity, sleep hygiene, stress management, smoking cessation, and limiting or avoiding alcohol.

The evidence base for “lifestyle as medicine” for improving physical health is well-established (Khaw et al., 2008; Ornish, 2009; James et al., 2013; Morton et al., 2014; Orlich and Fraser, 2014; An and Xiang, 2015; Dawber et al., 2015; Wright et al., 2017; Loprinzi and Joyner, 2018; Tan et al., 2018; Petrides et al., 2019), but the benefits of Lifestyle Medicine for mental health has only recently emerged (Velten et al., 2018; Kim et al., 2019; Riemann et al., 2020). A recent meta-analysis of 41 studies conducted by Firth et al. (2020b) reported that lifestyle modifications were effective for the prevention and treatment of mental health disorders. Further, positive mental health has a protective effect against unhealthy lifestyles, physical disease and social inequalities (Lange, 2018). Indeed, Lifestyle Medicine has not received its warranted attention in the psychological literature.

## Physical Activity

Of all lifestyle factors, physical activity is most recognized for conferring mental health benefits and there is growing literature to support this. A meta-analysis of 49 prospective cohort studies (*n* = 266,939) reported that higher physical activity levels resulted in decreased odds of developing future depression and had a protective effect against depression regardless of age, sex, and geographical region (Schuch et al., 2018). Several other meta-analyses and reviews have also concluded that physical activity is an effective intervention for the treatment of depression (Cooney et al., 2013; Silveira et al., 2013; Josefsson et al., 2014; Kvam et al., 2016), and studies have shown exercise therapy to be comparable to pharmacotherapy for depression (Cooney et al., 2013; Kvam et al., 2016). Lathia et al. (2017) reported that among a cohort of over 12,000 adults, physically active individuals reported higher levels of happiness and increased positive affect while exercising.

Alarmingly, physical activity levels have declined worldwide during COVID-19 (Bentlage et al., 2020; Chtourou et al., 2020). Tison et al. (2020) used smartphone accelerometer data from 455,000 individuals across 187 countries, found a 5.5% decrease in the mean number of steps taken in the 10 days after the pandemic declaration, and then a 27.3% decrease at 30 days. Considerable variability was reported between countries, with Italy recording the greatest decrease in physical activity of 48.5%. Further, the ECLB-COVID-19 international online survey reported that time spent sitting increased by 28% and physical activity decreased for all fitness levels (Ammar et al., 2020b). Clearly, physical activity should be frontline in COVID-19 targeted universal mental health interventions. The World Health Organization (2020a) recommends that adults achieve at least 150–300 minutes of moderate-intensity aerobic physical activity per week and 60 minutes per day for children and adolescents aged 5–17 years. For practical strategies for increasing physical activity refer to Table 1.

## Nutrition

There is an increasing interest in the connection between nutrition and mental health (Firth et al., 2020a). A meta-analysis of 21 epidemiological studies concluded that the Western diet, characterized by a high intake of red and/or processed meat, refined grains, sweets, and high fat dairy products is associated with a higher risk of depression (Li et al., 2017). Conversely, a healthy diet consisting of high intakes of vegetables, fruits, wholegrains, soy, fish, and low-fat dairy was found to be associated with a lower risk of depression. Extending these epidemiological observations, a recent meta-analysis of 15 RCTs (*n* = 45,826) also concluded that dietary interventions significantly reduced depressive symptoms across the population (Firth et al., 2019). Healthy eating patterns have also been associated with positive emotional states. In a systematic review of 10 studies (Tuck et al., 2019), consuming or exceeding the recommended amount of fruit and vegetables was associated with psychological well-being.

Mattioli et al. (2020) observed that the COVID-19 quarantine period was associated with poorer eating patterns. Of particular relevance is a decreased consumption of fruit and vegetables, which are rich sources of antioxidants and vitamins needed to help fight infections. Conversely, unhealthy diets may activate inflammatory processes that weaken the innate and adaptive immune systems (Butler and Barrientos, 2020). These findings align with the results of the ECLB-COVID19 international online survey which found eating unhealthy food, eating out of control, snacking between meals, and the number of main meals per day increased significantly (*p* < 0.001) during the pandemic. Interestingly, alcohol binging significantly decreased during the pandemic (Ammar et al., 2020b). In recognition of the COVID-19 mediated decline in diet quality (Ingram et al., 2020; Robinson et al., 2021), and the mental health and immune benefits associated with eating plant-based foods (Arshad et al., 2020), emphasis on positive nutrition is needed. For practical suggestions for a healthy diet refer to Table 1.

## Sleep

Sleep hygiene plays a crucial role in mental health and well-being. Loprinzi and Joyner (2018) conducted a prospective cohort study (*n* = 13,423) and found that attaining optimal levels of sleep of 7–9 h for adults, was associated with better Health-Related Quality of Life (HRQOL) and reduced premature mortality risk.

During COVID-19, sleep patterns and sleep quality has been adversely affected. In a survey of 6,041 Canadians, 77.8% reported disturbed sleep during the pandemic, with those 41–60 years of age being twice as likely to report sleep disturbances compared to those less than 25 years (Osiogo et al., 2021). An Italian study of 1,035 young adults under 35 years of age observed a shift in sleep-wake rhythms during lockdown, with people going to bed and waking up later (Cellini et al., 2020). The ECLB COVID-19 international study of 5,053 individuals in quarantine reported severe disruptions in sleep hygiene, although sleep quality during home confinement was not as affected among highly active individuals (Trabelsi et al., 2021). For practical recommendations to improve sleep hygiene refer to Table 1.

## Multicomponent Lifestyle Interventions

In the same way that there has been a trend toward multicomponent PPIs, lifestyle interventions are commonly delivered in a multicomponent fashion. For example, combined nutrition and physical activity interventions are commonplace for addressing weight loss and certain chronic conditions, such as type 2 diabetes (Cradock et al., 2017; Chater et al., 2020) and cardiovascular disease (Barbaresko et al., 2018). While research examining the relative effectiveness of multicomponent lifestyle interventions targeting mental health and well-being is scant and inconclusive (Gomez-Gomez et al., 2020), we hypothesis that the multicomponent approach would likely confer greater benefits than a single modality approach. Indeed, in the ECLB-COVID19 international survey, Ammar et al. (2020c) concluded that the adverse psychosocial impact of home quarantine was correlated with multiple unhealthy lifestyle behaviors and they called for the urgent deployment of interdisciplinary interventions that foster an active healthy confinement lifestyle (AHCL).

## A Call for Interdisciplinary Universal Mental Health Interventions

Historically, the disciplines of Lifestyle Medicine and Positive Psychology have functioned independently, but there is growing awareness of their complimentary nature. For example, a commonly cited well-being framework within the Positive Psychological literature is PERMA, an acronym asserted by Seligman a decade ago to encapsulate the components of a “flourishing life”: Positive emotion, Engagement, Relationships, Meaning and Achievement (Seligman, 2011). The PERMA model has subsequently been expanded to PERMA-H (Health) (Lai et al., 2018; Mayer, 2019), in recognition of the contribution of lifestyle behaviors to well-being. Similarly, within the Lifestyle Medicine discipline, which has historically focused on modifiable health behaviors for improving health outcomes, there has been the formalization of a “Positive Health” initiative (Seligman, 2008) which has the remit of embedding Positive Psychology principles into clinical practice. Hence, there is an increasing recognition that optimal health and well-being outcomes can be achieved through an interdisciplinary approach. Notably, in his seminal paper published one decade ago in American Psychologist, Walsh (2011) aptly identified interdisciplinary contributors to mental health and wellbeing, and advocated for greater attention to be placed on them. Indeed, in the midst and wake of the COVID-19 pandemic there has never been a greater need for a universal and interdisciplinary approach to disseminate effective therapeutic strategies that not only meets the mental health challenges faced by societies around the world, but also provides wholistic care. Incorporating physical, mental, social, and spiritual health are critical elements to cope with personal adversity, improve well-being, and reduce suffering and disease (Ammar et al., 2020a; Del Castillo, 2020; Ferrell et al., 2020; Lucchetti et al., 2020).

Accordingly, we argue that to optimize the effectiveness of mental health and well-being interventions, they should adopt an interdisciplinary approach, integrating evidence-based strategies from both the Positive Psychology and Lifestyle Medicine literature. The literature is scant in bringing these two disciplines together to improve mental health and well-being, although several studies (Morton et al., 2020; Przybylko et al., 2021; Renfrew et al., 2020) have suggested this interdisciplinary and multimodal approach may result in better outcomes than single modality interventions alone.

With many countries calling for population-level mental health and well-being solutions, the digital delivery of universal interdisciplinary mental health interventions offers a cost-effective and scalable way forward for promoting mental health, even given the limitations imposed by COVID-19, such as confinement, social isolation and the financial crisis. Innovation in digital technology has resulted in the ability to offer interventions that: involve fewer resources and personnel required for rollout and implementation; overcome geographic barriers and hence have a larger reach; are cheaper to disseminate; overcome issues of anonymity; and provide more flexibility for participants (Deady et al., 2017). In a recent review, Rauschenberg et al. (2020) concluded that eHealth interventions are well-suited to mitigate the adverse psychological effects of the COVID-19 pandemic at a population level, and encouraged the development of digital strategies for the development of mental health promotion, prevention and care. Similarly, Ammar et al. (2021) encouraged innovative digital approaches to track, predict and facilitate the user's adherence to AHCL. Further, Torous et al. (2020) argued that COVID-19 has presented an impetus to increase investment in digital health to provide high-quality mental health care for the future.

In the wake of the unprecedented mental health crisis caused by COVID-19, multiple calls have been made for novel and innovative mental health solutions (Firth et al., 2020b; Holmes et al., 2020). Rudd and Beidas (2020) called for a paradigm shift to include novel interventions that deliver mental health support for all people with a greater focus in the delivery of mental health prevention. Hence, we call for the creation and rollout of well-designed interdisciplinary universal mental health interventions, involving multiple evidence-based strategies from both Positive Psychology and Lifestyle Medicine, that can be provided cost-effectively at a population level through digital delivery in the wake of the COVID-19 pandemic and beyond.

The strength of this perspective article is that it argues for a novel approach, using universal interdisciplinary interventions that combine Positive Psychology and Lifestyle Medicine, for improving mental well-being. Further, it calls for the use of digital mental health solutions to achieve scalability and accessibility within the limitations of COVID-19 lockdown and restrictions. The main limitation of this perspective article is the scant research in the area of universal interdisciplinary interventions in the context of COVID-19.

## Conclusion

In this perspective article, interdisciplinary universal mental health interventions, delivered digitally, have been presented as a potential strategy for improving the mental health of populations during the COVID-19 pandemic and beyond. We argue that the application of universal interventions that combine evidenced-based strategies from the Positive Psychology and Lifestyle Medicine literature provides an innovative way to protect and buffer the mental well-being of populations. Future research could be undertaken to investigate an integrated mental healthcare model that incorporates universal interventions, clinical practice, and digital support.

## Data Availability Statement

The original contributions presented in the study are included in the article. Further inquiries can be directed to the corresponding author/s.

## Author Contributions

GP and DM conceptualized the work and ideated the structure. GP analyzed the literature and wrote the manuscript. All authors contributed to the revision and editing of the manuscript.

## Conflict of Interest

The authors declare that the research was conducted in the absence of any commercial or financial relationships that could be construed as a potential conflict of interest.

## References

[B1] Altena E. Baglioni C. Espie C. A. Ellis J. Gavriloff D. Holzinger B.. (2020). Dealing with sleep problems during home confinement due to the COVID-19 outbreak: Practical recommendations from a task force of the European CBT-I Academy. J. Sleep Res. 29:e13052. 10.1111/jsr.13052 32246787

[B2] Ammar A. Bouaziz B. Trabelsi K. Glenn J. Zmijewski P. Müller P.. (2021). Applying digital technology to promote active and healthy confinement lifestyle during pandemics in the elderly. Biol. Sport 38, 391–396. 10.5114/biolsport.2021.100149 PMC8329971 34475622

[B3] Ammar A. Brach M. Trabelsi K. Chtourou H. Boukhris O. Masmoudi L.. (2020b). Effects of COVID-19 home confinement on eating behaviour and physical activity: results of the ECLB-COVID19 international online survey. Nutrients 12:1583. 10.3390/nu12061583 32481594 PMC7352706

[B4] Ammar A. Mueller P. Trabelsi K. Chtourou H. Boukhris O. Masmoudi L.. (2020c). Psychological consequences of COVID-19 home confinement: The ECLB-COVID19 multicenter study. PLoS ONE 15:e0240204. 10.1371/journal.pone.0240204 33152030 PMC7643949

[B5] Ammar A. Trabelsi K. Brach M. Chtourou H. Boukhris O. Masmoudi L.. (2020a). Effects of home confinement on mental health and lifestyle behaviours during the COVID-19 outbreak: insight from the “ECLB-COVID19” multi countries survey. MedRxiv. 10.1101/2020.05.04.20091017 PMC7996377 33795912

[B6] An R. Xiang X. (2015). Smoking, heavy drinking, and depression among U.S. middle-aged and older adults. Prev. Med. 81, 295–302. 10.1016/j.ypmed.2015.09.026 26436684

[B7] Arshad M. S. Khan U. Sadiq A. Khalid W. Hussain M. H. Yasmeen A.. (2020). Coronavirus disease (CVOID-19) and immunity booster green foods: a mini review. Food Sci. Nutr. 8, 3971–3976. 10.1002/fsn3.1719 32837716 PMC7300634

[B8] Bae S. Kim S. R. Kim M. N. Shim W. J. Park S. M. (2021). Impact of cardiovascular disease and risk factors on fatal outcomes in patients with COVID-19 according to age: a systematic review and meta-analysis. Heart 107, 373–380. 10.1136/heartjnl-2020-317901 33334865

[B9] Barbaresko J. Rienks J. Nothlings U. (2018). Lifestyle indices and cardiovascular disease risk: a meta-analysis. Am. J. Prev. Med. 55, 555–564. 10.1016/j.amepre.2018.04.046 30241617

[B10] Bentlage E. Ammar A. How D. Ahmed M. Trabelsi K. Chtourou H.. (2020). Practical recommendations for maintaining active lifestyle during the COVID-19 pandemic: a systematic literature review. Int. J. Environ. Res. Public Health 17:6265. 10.3390/ijerph17176265 32872154 PMC7503956

[B11] Bueno-Notivol J. Gracia-Garcia P. Olaya B. Lasheras I. Lopez-Anton R. Santabarbara J. (2021). Prevalence of depression during the COVID-19 outbreak: a meta-analysis of community-based studies. Int. J. Clin. Health Psychol. 21:100196. 10.1016/j.ijchp.2020.07.007 32904715 PMC7458054

[B12] Butler M. J. Barrientos R. M. (2020). The impact of nutrition on COVID-19 susceptibility and long-term consequences. Brain Behav. Immun. 87, 53–54. 10.1016/j.bbi.2020.04.040 32311498 PMC7165103

[B13] Carr A. Cullen K. Keeney C. Canning C. Mooney O. Chinseallaigh E.. (2020). Effectiveness of positive psychology interventions: a systematic review and meta-analysis. J. Posit. Psychol. 1–21. 10.1080/17439760.2020.1818807

[B14] Cellini N. Canale N. Mioni G. Costa S. (2020). Changes in sleep pattern, sense of time and digital media use during COVID-19 lockdown in Italy. J. Sleep Res. 29:e13074. 10.1111/jsr.13074 32410272 PMC7235482

[B15] Chater A. M. Smith L. Ferrandino L. Wyld K. Bailey D. P. (2020). Health behaviour change considerations for weight loss and type 2 diabetes- nutrition, physical activity and sedentary behaviour. Pract. Diabetes 37, 228–231. 10.1002/pdi.2311

[B16] Chtourou H. Trabelsi K. H'Mida C. Boukhris O. Glenn J. M. Brach M.. (2020). Staying physically active during the quarantine and self-isolation period for controlling and mitigating the COVID-19 pandemic: a systematic overview of the literature. Front. Psychol. 11:1708. 10.3389/fpsyg.2020.01708 33013497 PMC7466737

[B17] CNN health (2021). Tracking Coronavirus' Global Spread. Available online at: https://edition.cnn.com/interactive/2020/health/coronavirus-maps-and-cases/

[B18] Cooney G. M. Dwan K. Greig C. A. Lawlor D. A. Rimer J. Waugh F. R.. (2013). Exercise for depression. Cochrane Database Syst. Rev. 9:CD004366. 10.1002/14651858.CD004366.pub6 PMC9721454 24026850

[B19] Cradock K. A. ÓLaighin G. Finucane F. M. Gainforth H. L. Quinlan L. R. Ginis K. A. (2017). Behaviour change techniques targeting both diet and physical activity in type 2 diabetes: A systematic review and meta-analysis. Int. J. Behav. Nutr. Phys. Act. 14:18. 10.1186/s12966-016-0436-0 28178985 PMC5299734

[B20] Cuartas J. (2020). Heightened risk of child maltreatment amid the COVID-19 pandemic can exacerbate mental health problems for the next generation. Psychol. Trauma 12, S195–S196. 10.1037/tra0000597 32463282

[B21] Cunha L. F. Pellanda L. C. Reppold C. T. (2019). Positive psychology and gratitude interventions: a randomized clinical trial. Front. Psychol. 10:584. 10.3389/fpsyg.2019.00584 30949102 PMC6437090

[B22] Curry O. S. Rowland L. A. Van Lissa C. J. Zlotowitz S. McAlaney J. Whitehouse H. (2018). Happy to help? A systematic review and meta-analysis of the effects of performing acts of kindness on the well-being of the actor. J. Exp. Soc. Psychol. 76, 320–329. 10.1016/j.jesp.2018.02.014

[B23] da Cunha de Sá-Caputo D. Taiar R. Seixas A. Sanudo B. Sonza A. Bernardo-Filho M. (2020). A proposal of physical performance tests adapted as home workout options during the COVID-19 pandemic. Appl. Sci. 10:4755. 10.3390/app10144755

[B24] Dawber T. R. Moore F. E. Mann G. V. (2015). II. Coronary Heart Disease in the Framingham Study. Int. J. Epidemiol. 44, 1767–1780. 10.1093/ije/dyv346 26705414

[B25] Deady M. Choi I. Calvo R. A. Glozier N. Christensen H. Harvey S. B. (2017). eHealth interventions for the prevention of depression and anxiety in the general population: a systematic review and meta-analysis. BMC Psychiatry 17:310. 10.1186/s12888-017-1473-1 28851342 PMC5576307

[B26] Del Castillo F. A. (2020). Health, spirituality and Covid-19: themes and insights. J. Public Health. 12:fdaa185. 10.1093/pubmed/fdaa185 33044544 PMC7665609

[B27] Deng J. Zhou F. Hou W. Silver Z. Wong C. Y. Chang O.. (2020). The prevalence of depression, anxiety, and sleep disturbances in COVID-19 patients: a meta-analysis. Ann. N. Y. Acad. Sci. 1486, 90–111. 10.1111/nyas.14506 33009668 PMC7675607

[B28] Dubey S. Biswas P. Ghosh R. Chatterjee S. Dubey M. J. Chatterjee S.. (2020). Psychosocial impact of COVID-19. Diabetes Metab. Syndr. 14, 779–788. 10.1016/j.dsx.2020.05.035 32526627 PMC7255207

[B29] Ettman C. K. Abdalla S. M. Cohen G. H. Sampson L. Vivier P. M. Galea S. (2020). Prevalence of depression symptoms in US adults before and during the COVID-19 pandemic. JAMA Netw. Open 3:e2019686. 10.1001/jamanetworkopen.2020.19686 32876685 PMC7489837

[B30] Evans A. Bufka L. (2020). The critical need for a population health approach: addressing the nation's behavioral health during the COVID-19 pandemic and beyond. Prev. Chronic Dis. 17:200261. 10.5888/pcd17.200261 32762806 PMC7417017

[B31] Ferrell B. R. Handzo G. Picchi T. Puchalski C. Rosa W. E. (2020). The urgency of spiritual care: COVID-19 and the critical need for whole-person palliation. J. Pain Symptom Manage. 60, e7–e11. 10.1016/j.jpainsymman.2020.06.034 32629084 PMC7332903

[B32] Firth J. Gangwisch J. E. Borisini A. Mayer E. (2020a). Food and mood: how do diet and nutrition affect mental wellbeing? Br. Med. J. 369:m2440. 10.1136/bmj.m2440 32601102 PMC7322666

[B33] Firth J. Marx W. Dash S. Carney R. Teasdale S. B. Solmi M.. (2019). The effects of dietary improvement on symptoms of depression and anxiety: a meta-analysis of randomized controlled trials. Psychosom. Med. 81, 265–280. 10.1097/PSY.0000000000000673 30720698 PMC6455094

[B34] Firth J. Solmi M. Wootton R. Vancampfort D. Schuch F. B. Hoare E.. (2020b). A meta-review of “lifestyle psychiatry”- the role of exercise, smoking, diet and sleep in the prevention and treatment of mental disorders. World Psychiatry 19, 360–380. 10.1002/wps.20773 32931092 PMC7491615

[B35] Fischer R. Bortolini T. Karl J. A. Zilberberg M. Robinson K. Rabelo A.. (2020). Rapid review and meta-meta-analysis of self-guided interventions to address anxiety, depression, and stress during COVID-19 social distancing. Front. Psychol. 11:563876. 10.3389/fpsyg.2020.563876 33192837 PMC7655981

[B36] Fisher J. R. Tran T. Hammerberg K. Sastry J. Nguyen H. Rowe H.. (2020). Mental health of people in Australia in the first month of COVID-19 restrictions: a national survey. Med. J. Aust. 213, 458–464. 10.5694/mja2.50831 33107063 PMC7756394

[B37] Ghielen S. T. S. van Woerkom M. Meyers M. C. (2017). Promoting positive outcomes through strengths interventions: a literature review. J. Posit. Psychol. 13, 573–585. 10.1080/17439760.2017.136516419933296

[B38] Gomez-Gomez I. Bellon J. A. Resurreccion D. M. Cuijpers P. Moreno-Peral P. Rigabert A.. (2020). Effectiveness of universal multiple-risk lifestyle interventions in reducing depressive symptoms: systematic review and meta-analysis. Prev. Med. 134:106067. 10.1016/j.ypmed.2020.106067 32194097

[B39] Greenberg M. T. Abenavoli R. (2016). Universal Interventions: fully exploring their impacts and potential to produce population-level impacts. J. Res. Educ. Eff. 10, 40–67. 10.1080/19345747.2016.1246632

[B40] Hendriks T. Schotanus-Dijkstra M. Hassankhan A. de Jong J. Bohlmeijer E. (2019). The efficacy of multi-component positive psychology interventions: a systematic review and meta-analysis of randomized controlled trials. J. Happiness Stud. 21, 357–390. 10.1007/s10902-019-00082-1

[B41] Holmes E. A. O'Connor R. C. Perry V. H. Tracey I. Wessely S. Arseneault L.. (2020). Multidisciplinary research priorities for the COVID-19 pandemic: a call for action for mental health science. Lancet Psychiatry 7, 547–560. 10.1016/s2215-0366(20)30168-1 32304649 PMC7159850

[B42] Ingram J. Maciejewski G. Hand C. J. (2020). Changes in diet, sleep, and physical activity are associated with differences in negative mood during COVID-19 lockdown. Front. Psychol. 11:588604. 10.3389/fpsyg.2020.588604 32982903 PMC7492645

[B43] James P. Troped P. J. Hart J. E. Joshu C. E. Colditz G. A. Brownson R. C.. (2013). Urban sprawl, physical activity, and body mass index: nurses' Health study and Nurses' Health study II. Am. J. Public Health 103, 369–375. 10.2105/AJPH.2011.300449 22698015 PMC3558772

[B44] Josefsson T. Lindwall M. Archer T. (2014). Physical exercise intervention in depressive disorders: meta-analysis and systematic review. Scand. J. Med. Sci. Sports 24, 259–272. 10.1111/sms.12050 23362828

[B45] Kavcic T. Avsec A. Zager Kocjan G. (2020). Psychological functioning of Slovene adults during the COVID-19 pandemic: Does resilience matter? Psychiatr. Q. 92, 207–216. 10.1007/s11126-020-09789-4 32556914 PMC7299145

[B46] Keyes K. M. Galea S. (2016). Setting the agenda for a new discipline: population Health Science. Am. J. Public Health 106, 633–634. 10.2105/AJPH.2016.303101 26959265 PMC4804350

[B47] Khaw K. T. Wareham N. Bingham S. Welch A. Luben R. Day N. (2008). Combined impact of health behaviours and mortality in men and women- The EPIC-Norfolk prospective population study. PLoS Med. 5:e12. 10.1371/journal.pmed.0050012 18184033 PMC2174962

[B48] Kim S. Y. Park J. H. Lee M. Y. Oh K. S. Shin D. W. Shin Y. C. (2019). Physical activity and the prevention of depression: a cohort study. Gen. Hosp. Psychiatry 60, 90–97. 10.1016/j.genhosppsych.2019.07.010 31376646

[B49] Kobau R. Seligman M. E. Peterson C. Diener E. Zack M. M. Chapman D.. (2011). Mental health promotion in public health: perspectives and strategies from positive psychology. Am. J. Public Health 101, e1–e9. 10.2105/AJPH.2010.300083 PMC3134513 21680918

[B50] Kvam S. Kleppe C. L. Nordhus I. H. Hovland A. (2016). Exercise as a treatment for depression: a meta-analysis. J. Affect. Disord. 202, 67–86. 10.1016/j.jad.2016.03.063 27253219

[B51] Lai M. K. Leung C. Kwok S. Y. C. Hui A. N. N. Lo H. H. M. Leung J. T. Y.. (2018). A multidimensional PERMA-H positive education model, general satisfaction of school life, and character strengths use in Hong Kong senior primary school students: confirmatory factor analysis and path analysis using the APASO-II. Front. Psychol. 9:1090. 10.3389/fpsyg.2018.01090 30008690 PMC6034423

[B52] Lange K. W. (2018). Diet, exercise, and mental disorders - Public health challenges of the future. Mov. Nutr. Health Dis. 2, 39–59. 10.5283/mnhd.12

[B53] Lathia N. Sandstrom G. M. Mascolo C. Rentfrow P. J. (2017). Happier people live more active lives: using smartphones to link happiness and physical activity. PLoS ONE 12:e0160589. 10.1371/journal.pone.0160589 28052069 PMC5213770

[B54] Li Y. Lv M. R. Wei Y. J. Sun L. Zhang J. X. Zhang H. G.. (2017). Dietary patterns and depression risk: a meta-analysis. Psychiatry Res. 253, 373–382. 10.1016/j.psychres.2017.04.020 28431261

[B55] Loades M. E. Chatburn E. Higson-Sweeney N. Reynolds S. Shafran R. Brigden A.. (2020). Rapid systematic review: the impact of social isolation and loneliness on the mental health of children and adolescents in the context of COVID-19. J. Am. Acad. Child Adolesc. Psychiatry 59, 1218–1239. 10.1016/j.jaac.2020.05.009 32504808 PMC7267797

[B56] Loprinzi P. D. Joyner C. (2018). Meeting sleep guidelines is associated with better health-related quality of life and reduced premature all-cause mortality risk. Am. J. Health Promot. 32, 68–71. 10.1177/0890117116687459 29214822

[B57] Lucchetti G. Goes L. G. Amaral S. G. Ganadjian G. T. Andrade I. Almeida P. O. A.. (2020). Spirituality, religiosity and the mental health consequences of social isolation during Covid-19 pandemic. Int. J. Soc. Psychiatry. 10.1177/0020764020970996 33135559 PMC7649649

[B58] Mattioli A. V. Sciomer S. Cocchi C. Maffei S. Gallina S. (2020). Quarantine during COVID-19 outbreak: changes in diet and physical activity increase the risk of cardiovascular disease. Nutr. Metab. Cardiovasc. Dis. 30, 1409–1417. 10.1016/j.numecd.2020.05.020 32571612 PMC7260516

[B59] Mayer S. C. (2019). Wellbeing in the workplace: a comprehensive model and best-practices from top-employers: adaption of PERMA (H) (Doctoral dissertation). Available online at: https://repositorio.iscte-iul.pt/handle/10071/19676

[B60] Mongrain M. Anselmo-Matthews T. (2012). Do positive psychology exercises work? A replication of Seligman et al. (2005). J. Clin. Psychol. 68, 382–389. 10.1002/jclp.21839 24469930

[B61] Morton D. Hinze J. Craig B. Herman W. Kent L. Beamish P.. (2020). A multimodal intervention for improving the mental health and emotional well-being of college students. Am. J. Lifestyle Med. 14, 216–224. 10.1177/1559827617733941 32231487 PMC7092406

[B62] Morton D. Rankin P. Kent L. Sokolies R. Dysinger W. Gobble J.. (2014). The Complete Health Improvement Program (CHIP) and reduction of chronic disease risk factors in Canada. Can. J. Diet. Pract. Res. 75, 72–77. 10.3148/75.2.2014.72 24897012

[B63] Orlich M. J. Fraser G. E. (2014). Vegetarian diets in the Adventist Health Study 2: a review of initial published findings. Am. J. Clin. Nutr. 100(Suppl. 1), 353S−358S. 10.3945/ajcn.113.071233 24898223 PMC4144107

[B64] Ornish D. (2009). Intensive lifestyle changes and health reform. Lancet Oncol. 10, 638–639. 10.1016/S14702045(09)70175-5 19573793

[B65] Osiogo F. Shalaby R. Adegboyega S. Hrabok M. Gusnowski A. Vuong W.. (2021). COVID-19 pandemic: demographic and clinical correlates of disturbed sleep among 6,041 Canadians. Int. J. Psychiatry Clin. Pract. 1–8. 10.1080/13651501.2021.1881127 33606597

[B66] Palmer K. Monaco A. Kivipelto M. Onder G. Maggi S. Michel J. P.. (2020). The potential long-term impact of the COVID-19 outbreak on patients with non-communicable diseases in Europe: consequences for healthy ageing. Aging Clin. Exp. Res. 32, 1189–1194. 10.1007/s40520-020-01601-4 32458356 PMC7248450

[B67] Pera A. (2020). Cognitive, behavioral, and emotional disorders in populations affected by the COVID-19 outbreak. Front. Psychol. 11:2263. 10.3389/fpsyg.2020.02263 33041902 PMC7525188

[B68] Petrides J. Collins P. Kowalski A. Sepede J. Vermeulen M. (2019). Lifestyle changes for disease prevention. Prim. Care 46, 1–12. 10.1016/j.pop.2018.10.003 30704651

[B69] Prinzing M. M. Zhou J. West T. N. Le Nguyen K. D. Wells J. C. Fredrickson B. L. (2020). Staying ‘In Sync’ with others during COVID-19-positivity resonance mediates cross-sectional and longitudinal links between trait resilience and mental health. J. Pos. Psychol. 10.1080/17439760.2020.1858336

[B70] Przybylko G. Morton D. P. Morton J. K. Renfrew M. E. Hinze J. (2021). An interdisciplinary mental wellbeing intervention for increasing flourishing: two experimental studies. J. Pos. Psychol. 1–16. 10.1080/17439760.2021.1897868

[B71] Purtle J. Nelson K. L. Counts N. Z. Yudell M. (2020). Population-based approaches to mental health: history, strategies, and evidence. Annu. Rev. Public Health 41, 201–221. 10.1146/annurev-publhealth-040119-094247 31905323 PMC8896325

[B72] Rauschenberg C. Schick A. Hirja D. Seidler A. Apfelbacher C. Riedel-Heller S.. (2020). Digital interventions to mitigate the negative impact of the COVID-19 pandemic on public mental health: a rapid meta-review. PsyArXiv. 10.31234/osf.io/uvc78 33606657 PMC7951054

[B73] Renfrew M. E. Morton D. P. Morton J. K. Hinze J. S. Beamish P. J. Przybylko G.. (2020). A web- and mobile app–based mental health promotion intervention comparing email, short message service, and videoconferencing support for a healthy cohort: randomized comparative study. J. Med. Internet Res. 22:e15592. 10.2196/15592 31904578 PMC6971514

[B74] Riemann D. Krone L. B. Wulff K. Nissen C. (2020). Sleep, insomnia, and depression. Neuropsychopharmacology 45, 74–89. 10.1038/s41386-019-0411-y 31071719 PMC6879516

[B75] Robinson E. Boyland E. Chisholm A. Harrold J. Maloney N. G. Marty L.. (2021). Obesity, eating behavior and physical activity during COVID-19 lockdown: a study of UK adults. Appetite 156:104853. 10.1016/j.appet.2020.104853 33038479 PMC7540284

[B76] Rossi R. Socci V. Pacitti F. Di Lorenzo G. Di Marco A. Siracusano A.. (2020). Mental health outcomes among frontline andsecond-line health care workers during the coronavirus disease 2019 (COVID-19) Pandemic in Italy. JAMA Netw. Open 3:e2010185. 10.1001/jamanetworkopen.2020.10185 32463467 PMC7256664

[B77] Rudd B. N. Beidas R. S. (2020). Digital mental health: the answer to the global mental health crisis? J. Med. Intern. Res. Ment. Health 7:e18472. 10.2196/18472 32484445 PMC7298632

[B78] Rusk R. D. Waters L. E. (2013). Tracing the size, reach, impact, and breadth of positive psychology. J. Posit. Psychol. 8, 207–221. 10.1080/17439760.2013.777766

[B79] Sampson L. Galea S. (2018). An argument for thefoundations of population mental health. Front. Psychiatry 9:600. 10.3389/fpsyt.2018.00600 30510524 PMC6252354

[B80] Santabarbara J. Lasheras I. Lipnicki D. M. Bueno-Notivol J. Perez-Moreno M. Lopez-Anton R.. (2020). Prevalence of anxiety in the COVID-19 pandemic: an updated meta-analysis of community-based studies. Prog. Neuropsychopharmacol. Biol. Psychiatry 109:110207. 10.1016/j.pnpbp.2020.110207 33338558 PMC7834650

[B81] Santarone K. McKenney M. Elkbuli A. (2020). Preserving mental health and resilience in frontline healthcare workers during COVID-19. Am. J. Emerg. Med. 38, 1530–1531. 10.1016/j.ajem.2020.04.030 32336584 PMC7156943

[B82] Schuch F. B. Vancampfort D. Firth J. Rosenbaum S. Ward P. B. Silva E. S.. (2018). Physical activity and incident depression: a meta-analysis of prospective cohort studies. Am. J. Psychiatry 175, 631–648. 10.1176/appi.ajp.2018.17111194 29690792

[B83] Seligman M. E. (2008). Positive health. Appl. Psychol. 57, 3–18. 10.1111/j.1464-0597.2008.00351.x

[B84] Seligman M. E. (2011). Flourish: A New Understanding of Happiness, Well-Being - and How to Achieve Them. Boston, MA: Nicholas Brealey.

[B85] Seligman M. E. Steen T. A. Park N. Peterson C. (2005). Positive psychology progress: empirical validation of interventions. Am. Psychol. 60, 410–421. 10.1037/0003-066X.60.5.410 16045394

[B86] Seligman M. E. P. (2019). Positive psychology: a personal history. Annu. Rev. Clin. Psychol. 15, 1–23. 10.1146/annurev-clinpsy-050718-095653 30525996

[B87] Sharma H. Verma S. (2020). Preservation of physical and mental health amid COVID-19 pandemic: recommendations from the existing evidence of disease outbreaks. Int. J. Acad. Med. 6, 76–82. 10.4103/IJAM.IJAM_47_20

[B88] Shi L. Lu Z. A. Que J. Y. Huang X. L. Liu L. Ran M. S.. (2020). Prevalence of and risk factors associated with mental health symptoms among the general population in China during the coronavirus disease 2019 pandemic. JAMA Netw. Open 3:e2014053. 10.1001/jamanetworkopen.2020.14053 32609353 PMC7330717

[B89] Silveira H. Moraes H. Oliveira N. Coutinho E. S. Laks J. Deslandes A. (2013). Physical exercise and clinically depressed patients: a systematic review and meta-analysis. Neuropsychobiology 67, 61–68. 10.1159/000345160 23295766

[B90] Sin N. L. Lyubomirsky S. (2009). Enhancing well-being and alleviating depressive symptoms with positive psychology interventions: a practice-friendly meta-analysis. J. Clin. Psychol. 65, 467–487. 10.1002/jclp.20593 19301241

[B91] Tan L. Wang M.-J. Modini M. Joyce S. Mykletun A. Christensen H.. (2014). Preventing the development of depression at work: a systematic review and meta-analysis of universal interventions in the workplace. BMC Med. 12:74. 10.1186/1741-7015-12-74 24886246 PMC4014627

[B92] Tan S. L. Storm V. Reinwand D. A. Wienert J. de Vries H. Lippke S. (2018). Understanding the positive associations of sleep, physical activity, fruit and vegetable intake as predictors of quality of life and subjective health across age groups: a theory based, cross-sectional web-based study. Front. Psychol. 9:977. 10.3389/fpsyg.2018.00977 29967588 PMC6016042

[B93] Tison G. H. Avram R. Abreau S. Kuhar P. Abreau S. Marcas G. M.. (2020). Worldwide effect of COVID-19 on physical activity: a descriptive study. Ann. Intern. Med. 173, 767–770. 10.7326/M20-2665 32598162 PMC7384265

[B94] Torous J. Jan Myrick K. Rauseo-Ricupero N. Firth J. (2020). Digital mental health and COVID-19: using technology today to accelerate the curve on access and quality tomorrow. J. Ment. Intern. Res. Ment. Health 7:e18848. 10.2196/18848 32213476 PMC7101061

[B95] Trabelsi K. Ammar A. Masmoudi L. Boukhris O. Chtourou H. Bouaziz B.. (2021). Globally altered sleep patterns and physical activity levels by confinement in 5056 individuals: ECLB COVID-19 international online survey. Biol. Sport 38, 495–506. 10.5114/biolsport.2021.101605 PMC8670812 34937958

[B96] Tsamakis K. Tsiptsios D. Ouranidis A. Mueller C. Schizas D. Terniotis C.. (2021). COVID-19 and its consequences on mental health (Review). Exp. Ther. Med. 21:244. 10.3892/etm.2021.9675 33603852 PMC7851613

[B97] Tuck N.-J. Farrow C. Thomas J. M. (2019). Assessing the effects of vegetable consumption on the psychological health of healthy adults- a systematic review of prospective research. Am. J. Clin. Nutr. 110, 196–211. 10.1093/ajcn/nqz080 31152539

[B98] United Nations (2020). Policy-Brief: COVID-19 and the Need for Action on Mental Health. Available online at: https://unsdg.un.org/resources/policy-brief-covid-19-and-need-action-mental-health

[B99] Velten J. Bieda A. Scholten S. Wannemuller A. Margraf J. (2018). Lifestyle choices and mental health: a longitudinal survey with German and Chinese students. BMC Public Health 18:632. 10.1186/s12889-018-5526-2 29769115 PMC5956886

[B100] Wade N. G. Hoyt W. T. Kidwell J. E. Worthington E. L. (2014). Efficacy of psychotherapeutic interventions to promote forgiveness: a meta-analysis. J. Consult. Clin. Psychol. 82, 154–170. 10.1037/a0035268 24364794

[B101] Walsh R. (2011). Lifestyle and mental health. Am. Psychol. 66, 579–592. 10.1037/a0021769 21244124

[B102] Waters L. Algoe S. B. Dutton J. Emmons R. Fredrickson B. L. Heaphy E.. (2021). Positive psychology in a pandemic: buffering, bolstering, and building mental health. J. Posit. Psychol. 1–21. 10.1080/17439760.2021.1871945

[B103] Wellenzohn S. Proyer R. T. Ruch W. (2016). Humor-based online positive psychology interventions: a randomized placebo-controlled long-term trial. J. Posit. Psychol. 11, 584–594. 10.1080/17439760.2015.1137624

[B104] World Health Organization (2017). Depression and Other Common Mental Disorders: Global Health Estimates. Available online at: http://apps.who.int/iris/bitstream/10665/254610/1/WHO-MSD-MER-2017.2-eng.pdf

[B105] World Health Organization (2020a). Physcial Activity. Available online at: https://www.who.int/news-room/fact-sheets/detail/physical-activity

[B106] World Health Organization (2020b). #HealthyAtHome - Physcial Activity. Available online at: https://www.who.int/news-room/campaigns/connecting-the-world-to-combat-coronavirus/healthyathome/healthyathome---physical-activity

[B107] Wright N. Wilson L. Smith M. Duncan B. McHugh P. (2017). The BROAD study: a randomised controlled trial using a whole food plant-based diet in the community for obesity, ischaemic heart disease or diabetes. Nutr. Diabetes 7:e256. 10.1038/nutd.2017.3 28319109 PMC5380896

[B108] Zheng Z. Peng F. Xu B. Zhao J. Liu H. Peng J.. (2020). Risk factors of critical and mortal COVID-19 cases: a systematic literature review and meta-analysis. J. Infect. 81, e16–e25. 10.1016/j.jinf.2020.04.021 32335169 PMC7177098

